# Considering Intrauterine Location in a Model of Fetal Growth Restriction After Maternal Titanium Dioxide Nanoparticle Inhalation

**DOI:** 10.3389/ftox.2021.643804

**Published:** 2021-03-23

**Authors:** J. N. D'Errico, S. B. Fournier, P. A. Stapleton

**Affiliations:** ^1^Department of Pharmacology and Toxicology, Ernest Mario School of Pharmacy, Rutgers University, Piscataway, NJ, United States; ^2^Environmental and Occupational Health Sciences Institute, Piscataway, NJ, United States

**Keywords:** fetal growth restriction, intrauterine position, titanium dioxide nanoparticle, spraque-dawley rat, rodent model, maternal exposures

## Abstract

Fetal growth restriction (FGR) is a condition with several underlying etiologies including gestational disease (e.g., preeclampsia, gestational diabetes) and xenobiotic exposure (e.g., environmental contaminants, pharmaceuticals, recreational drugs). Rodent models allow study of FGR pathogenesis. However, given the multiparous rodent pregnancy, fetal growth variability within uterine horns may arise. To ascertain whether intrauterine position is a determinant of fetal growth, we redesigned fetal weight analysis to include litter size and maternal weight. Our FGR model is produced by exposing pregnant Sprague Dawley rats to aerosolized titanium dioxide nanoparticles at 9.44 ± 0.26 mg/m^3^ on gestational day (GD) 4, GD 12 or GD 17 or 9.53 ± 1.01 mg/m^3^ between GD 4-GD 19. In this study fetal weight data was reorganized by intrauterine location (i.e., right/left uterine horn and ovarian/middle/vaginal position) and normalized by maternal weight and number of feti per uterine horn. A significant difference in fetal weight in the middle location in controls (0.061 g ± 0.001 vs. 0.055 g ± 0.002), GD 4 (0.033 g ± 0.003 vs. 0.049 g ± 0.004), and GD 17 (0.047 g ± 0.002 vs. 0.038 g ± 0.002) exposed animals was identified. Additionally, GD 4 exposure produced significantly smaller feti in the right uterine horn at the ovarian end (0.052 g ± 0.003 vs. 0.029 g ± 0.003) and middle of the right uterine horn (0.060 g ± 0.001 vs. 0.033 g ± 0.003). GD 17 exposure produced significantly smaller feti in the left uterine horn middle location (0.055g ± 0.002 vs. 0.033 ± 0.002). Placental weights were unaffected, and placental efficiency was reduced in the right uterine horn middle location after GD 17 exposure (5.74 g ± 0.16 vs. 5.09 g ± 0.14). These findings identified: (1) differences in fetal weight of controls between the right and left horns in the middle position, and (2) differential effects of single whole-body pulmonary exposure to titanium dioxide nanoparticles on fetal weight by position and window of maternal exposure. In conclusion, these results indicate that consideration for intrauterine position, maternal weight, and number of feti per horn provides a more sensitive assessment of FGR from rodent reproductive and developmental studies.

## Introduction

Fetal growth restriction (FGR) is a pathology where the full *in utero* growth potential for a fetus is not met during the period of gestation (Wollmann, 1998). In the clinical terms, this is identified as birth weight below the 10th percentile on a singleton growth curve (Bamfo and Odibo, 2011). Concerningly, a FGR diagnosis is commonly accompanied by other immediate health concerns of perinatal death and neonatal complications. Surviving newborns face an increased likelihood of developing cardiovascular disease, asthma, type 2 diabetes, and metabolic disorders later in life (Hales et al., 1991; Martyn et al., 1995; Curhan et al., 1996; Leon et al., 1998; Barker, 2006; Xu et al., 2014). Because of these associations with short- and long-term health conditions, FGR is a condition of concern for obstetric, pediatric, and primary care practitioners as well as reproductive and developmental scientists. Although a large body of work has identified several genetic and environmental etiologies of FGR, it is clear that many have yet to be uncovered (Wollmann, 1998; Sharma et al., 2016). Recently, accumulating evidence has suggested that pregnant women environmentally exposed to components of air pollution (e.g., fine/ultrafine fractions of particulate matter) or occupationally exposed to nanoparticles are at increased risk for FGR (Rogers and Dunlop, 2006; Manangama et al., 2019; Bekkar et al., 2020).

Laboratory studies have helped to unveil the molecular mechanisms and potential therapeutics of common gestational diseases and disorders. These may include models of preeclampsia, gestational diabetes, genetic abnormalities, infections, and maternal environmental exposures (e.g., high altitude, phthalates, heavy metals, and ultrafine particles), each likely to result in the development of FGR (Yamashita et al., 2003; Arce et al., 2012; Tunster et al., 2014; Xu et al., 2016; Shen et al., 2017; Bailey et al., 2019; Morales-Rubio et al., 2019; Tachibana et al., 2019; Chen et al., 2020). It is estimated that 95% of the animals used in these studies are multiparous rodents, including mice and rats (Vandamme, 2014). While these models have proven beneficial for understanding FGR and developmental and reproductive science, the nature of rodent uterine anatomy and placentation has inherent differences from humans.

A cornerstone of Developmental and Reproductive Toxicology (DART) studies is fetal weight evaluation. This involves calculating the average weight of a litter per dam, and subsequently averaging litter weights per experimental group, which for our purposes we will refer to as the “traditional” approach (Lazic and Essioux, 2013). This traditional approach may detect a significant impact on fetal body weight, but without consideration for variability between feti in specific uterine locations, right and left uterine horns, litter size, or maternal body weight. Studies utilizing a fetal pig model have shown that there is intra-litter variability in fetal growth, in that implantation in the middle of a uterine horn produces smaller feti than those deposited at the ends of the uterine horn (e.g., near the ovary or vagina) (Perry and Rowell, 1969; Jang et al., 2014). Furthermore, human studies identify that feti which implant on the lateral aspects of the uterus are more likely to be smaller and are at a higher risk for the development of preeclampsia, miscarriage, or preterm birth compared with feti that implant in anterior or posterior positionings (Gonser et al., 1996; Kalanithi et al., 2007; Magann et al., 2007). Particularly hazardous implantation sites that are low in the uterine body can result in placental growth that partially or entirely covers the cervix, a condition known as placenta previa (Balayla et al., 2019). The uterine tissue around the cervix does not have a strong blood supply and is not well-perfused; therefore, fetal implantations diagnosed with placenta previa are at heightened risk for placental ischemia, placental hypoxia, and reduced fetal growth (Harper et al., 2010). This evidence that implantation site is a factor in fetal growth underscores a need to account for intrauterine position in laboratory DART studies, especially when considering pregnancies that result in multiple offspring. Unfortunately, few rodent investigations have examined whether intrauterine position influences fetal body weight at term, thereby contributing to study variability.

The number of feti in a litter is negatively correlated with birth weight (Romero et al., 1992; Ishikawa et al., 2006; McLaurin and Mactutus, 2015). Romero and colleagues reported an inverse relationship between fetal weight and litter size in Sprague Dawley (SD) rats, and emphasized that if this relationship is not taken into account when toxicity on fetal weight is analyzed there is a potential to mask a decrease in fetal weight due to litter size reduction (Romero et al., 1992). Importantly, the number of feti deposited within the right and left uterine horns, respectively, not total litter size, determines the influence of the intrauterine position effect; therefore, experimental outcomes should be evaluated on a per-horn basis (Raz et al., 2012; McLaurin and Mactutus, 2015). Lastly, maternal weight may be an important consideration, as smaller dams may have less energy reserve per fetus to produce feti of a similar weight compared to a larger female (Thame et al., 2015).

The impact of environmental exposure during pregnancy affecting intrauterine positional growth has received little attention. Our model of maternal exposure to titanium dioxide nanoparticle (nano-TiO_2_) aerosols during gestation has identified impairments in gestational health (Stapleton et al., 2013; Fournier et al., 2019). Utilizing this model, we have previously reported effects on fetal reabsorption and placental and fetal weight depending on the window(s) of gestational exposure (Stapleton et al., 2013; Fournier et al., 2019). Therefore, the purpose of this study was to develop a stepwise method of analysis to organize and normalize fetal growth to more accurately assess FGR in our model. We evaluated fetal weight in a gravid SD rat model under control conditions and after maternal exposure to nano-TiO_2_ aerosols by position, maternal GD 20 weight, and number of feti per uterine horn. To ascertain whether exposure caused FGR in a particular uterine location and if the timing of the exposure generated a different outcome, the analysis was conducted with data from four different exposure scenarios: single exposure on gestational day (GD) 4, GD 12, or GD 17, and repeat exposures occurring between GD 4 and GD 19. This challenges the traditional methods and serves useful for future investigations of fetal growth.

## Methods

### Animals

Timed-pregnant SD rats were purchased from Charles River Laboratories (Kingston, NY). Animals were single housed in an AAALAC-approved Rutgers University vivarium where they were allowed access to Purina 5053 chow and water *ad libitum*. Animals arrived on GD 2 or GD 3. After a 24-h acclamation period, animals were randomly assigned to control (*n* = 21), filtered-air control (*n* = 6), or nano-TiO_2_ exposed groups (*n* = 6–9). Nano-TiO_2_ groups received a single exposure on GD 4, GD 12, or GD 17 as described previously (Fournier et al., 2019) or repeat exposures between GD 4 and GD 19 to aerosolized TiO_2_ nanoparticles via whole-body inhalation. Control animals were exposed to filtered air via whole-body inhalation. Naïve animals were not subjected to the inhalation facility. All procedures were approved by the Institutional Animal Care and Use Committee at Rutgers University.

### Particle Characterization

Powdered titanium dioxide was purchased from Evonik (Aeroxide TiO_2_, Parsippany, NJ). Previous characterization of the material determined a composition to be primarily anatase (80%) and rutile (20%) (Fournier et al., 2019). Primary particle size was determined to be 21 nm with a mean surface area of 48.08 mg^2^/g (Fournier et al., 2019).

### Inhalation Exposure to Aerosolized Titanium Dioxide Nanoparticles

The nano-TiO_2_ preparation and aerosol exposure has been detailed from one other study previously published (Fournier et al., 2019). Briefly, animals were administered whole-body inhalation exposures in a custom rodent inhalation facility (IEStechno, Morgantown, WV). The size distribution and relative mass of the particle aerosols were monitored in real time with a Scanning Mobility Particle Sizer (SMPS, TSI, Shoreview, MN). Aerosols were collected on a 47-nm PTFE membrane filter for gravimetric sampling to confirm concentration. The exposures were carried out for 4-hr on single days (i.e., GD 4, GD 12, GD 17) or took place repeatedly 5 days/weeks from GD 4 through GD 19. Average aerosol concentrations were measured at 9.44 ± 0.26 mg/m^3^ and 9.53 ± 1.01 mg/m^3^ for 4-h single and repeat exposures, respectively.

### Cesarean Procedure and Data Collection

On GD 20 animals were anesthetized via isoflurane inhalation (i.e., 5% induction, 3% maintenance) and positioned supine. Surgical scissors were used to create a Y-shaped incision through the abdomen to expose the uterus. The left and right gravid uterine horns were identified, removed, and individually pinned to a dissecting dish positioned with the ovary to the left and the vaginal end to the right.

**Figure 1:**
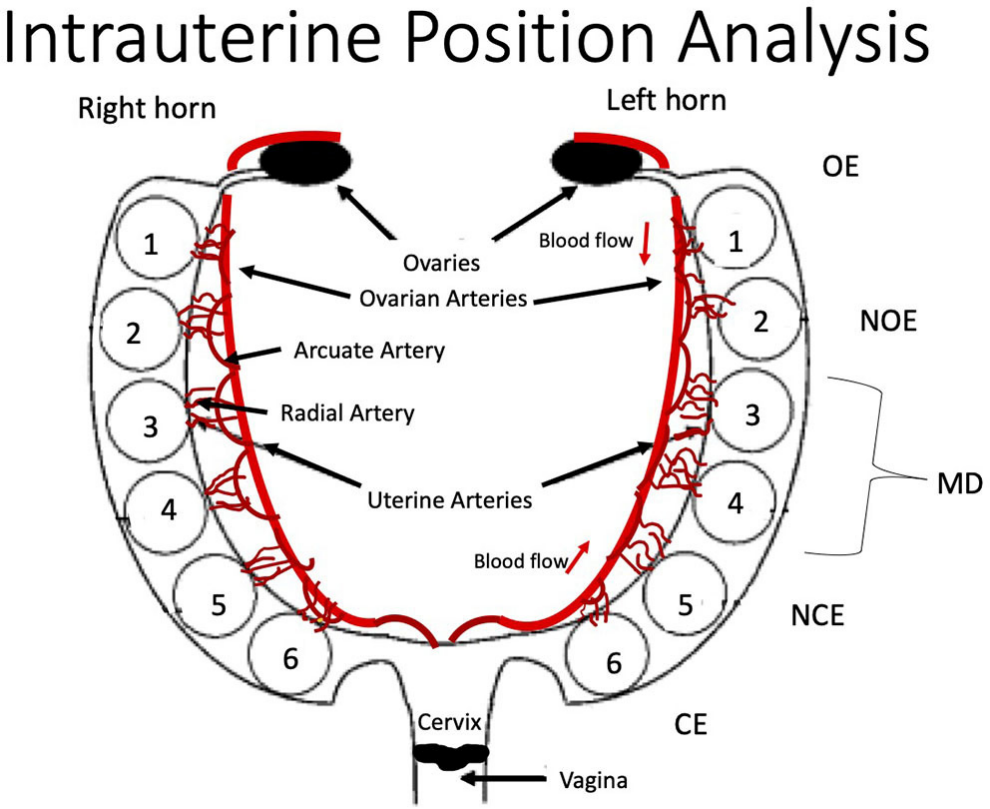
Schematic of rodent uterine horn anatomy from the ventral aspect and acronym key. Feti are numbered within each horn from the ovary end (OE) as fetus one to the cervical end (CE) as fetus six. Anatomy of major uterine and ovarian arteries and associated arcuate and radial artery branching is displayed. OE, Ovarian End; NOE, Next-to Ovarian End; MD, Middle; NCE, Next-to Cervical End; CE, Cervical End.

Using a surgical scissor, the uterine muscle was cut lengthwise to reveal placentas, amniotic sacs, and feti. The fetus from the ovary-most end was designated as fetus number one (Figure 1). Moving toward the vaginal end, each fetus was numbered, removed from its amniotic sac, and weighed. Associated placentas were weighed, and wet weights were recorded.

### Protocol for Intrauterine Position Analysis

The inclusion criteria in this analysis required a uterine horn to have at least five feti. Dams that had <5 feti in the litter were excluded from analysis (Table 1). Right or left uterine horns that had <5 feti were also excluded from analysis. Data from horns that met inclusion criteria were organized by intrauterine position [i.e., right/left ovary end (R/L OE), right/left next to ovary end (R/L NOE), right/left middle (R/L MD), right/left next to cervical end (R/L NCE) and right/left cervical end (R/L CE)] (Figure 1). Feti in the middle of uterine horns with more than five feti were averaged for the “middle” category.

**Table 1:** Reported number of litters and uterine horns excluded from analysis and older analyses of fetal weight demonstrating per dam and per treatment group averages along with percent reduction in fetal weight.

	**Naïve control**	**Repeat**	**GD4**	**GD12**	**GD17**
No. litters excluded	0/20	0/9	0/6	1/7	0/7
No. right horns excluded	5/20	1/9	2/6	3/7	0/7
No. left horns excluded	8/20	1/9	1/6	3/7	1/7
Fetal weight average per dam, per treatment group	2.62 ± 0.04	2.53 ± 0.05	2.49 ± 0.08	2.63 ± 0.07	2.57 ± 0.08
% decrease in fetal weight		4%	5%	0%	2%

Existing literature has shown that maternal horn size and litter size can influence the intrauterine position effect in the Sprague Dawley rat (Raz et al., 2012; McLaurin and Mactutus, 2015). To correct for differences in maternal weight and litter size, each fetal weight was normalized to the number of feti within that horn by the following equation:

$$\begin{array}{l} \text{Fetal Weight}/\left(\text{Maternal GD }20\text{ Weight}/\text{Number of Feti in Horn}\right) \end{array}$$

### Calculation of Placental Efficiency

Placental efficiency, a ratio of fetal weight to placental weight, is often used as a proxy measurement of placental function in human and laboratory animal studies (Hayward et al., 2016). Placental efficiency was calculated by dividing the raw fetal weight by its respective raw placental weight that were measured on GD 20.

### Statistical Analysis

A Grubb's test was employed to remove any outliers from the data set before further analysis. Student's *t*-test was then used to compare fetal weights using the traditional approach. Fetal weights were also analyzed by arranging and normalizing according to our developed protocol, the individual intrauterine positions from each group were compared between the right and left horn using a two-way ANOVA and Sidak's multiple comparisons test. The nano-TiO_2_ exposed group was compared with controls by position for the right and left horn using a two-way ANOVA and Sidak's multiple comparison test. Placental weights and placental efficiencies, calculated as a ratio of fetal weight to placental weight, were evaluated with a similar approach using raw unnormalized values. Effect sizes were calculated and included as Supplementary Table 1. Statistical analysis was conducted with GraphPad Prism 8.0 (San Diego, CA, USA). Data is reported as mean ± standard error. Statistical significance was set to *p* < 0.05 and is indicated with an asterisk (^*^).

## Results

### Traditional Analysis of Fetal Weight

Fetal body weight was compared between control dams exposed to filtered air in our inhalation exposure facility and naïve animal that never entered the facility using both the traditional and developed approaches. As in previous assessments, no statistical differences were observed (data not shown) between control filtered air exposed or naïve groups (Fournier et al., 2019). Therefore, in this study, all assessments compared each nano-TiO_2_ exposed group to a group naïve animals identified as “control.”

Fetal body weights were compared using the traditional approach of averaging fetal weight by litter and then by treatment group (Table 1). No significant differences and thus no FGR was found using this approach.

### Intrauterine Position on Fetal Body Weight

**Figure 2:**
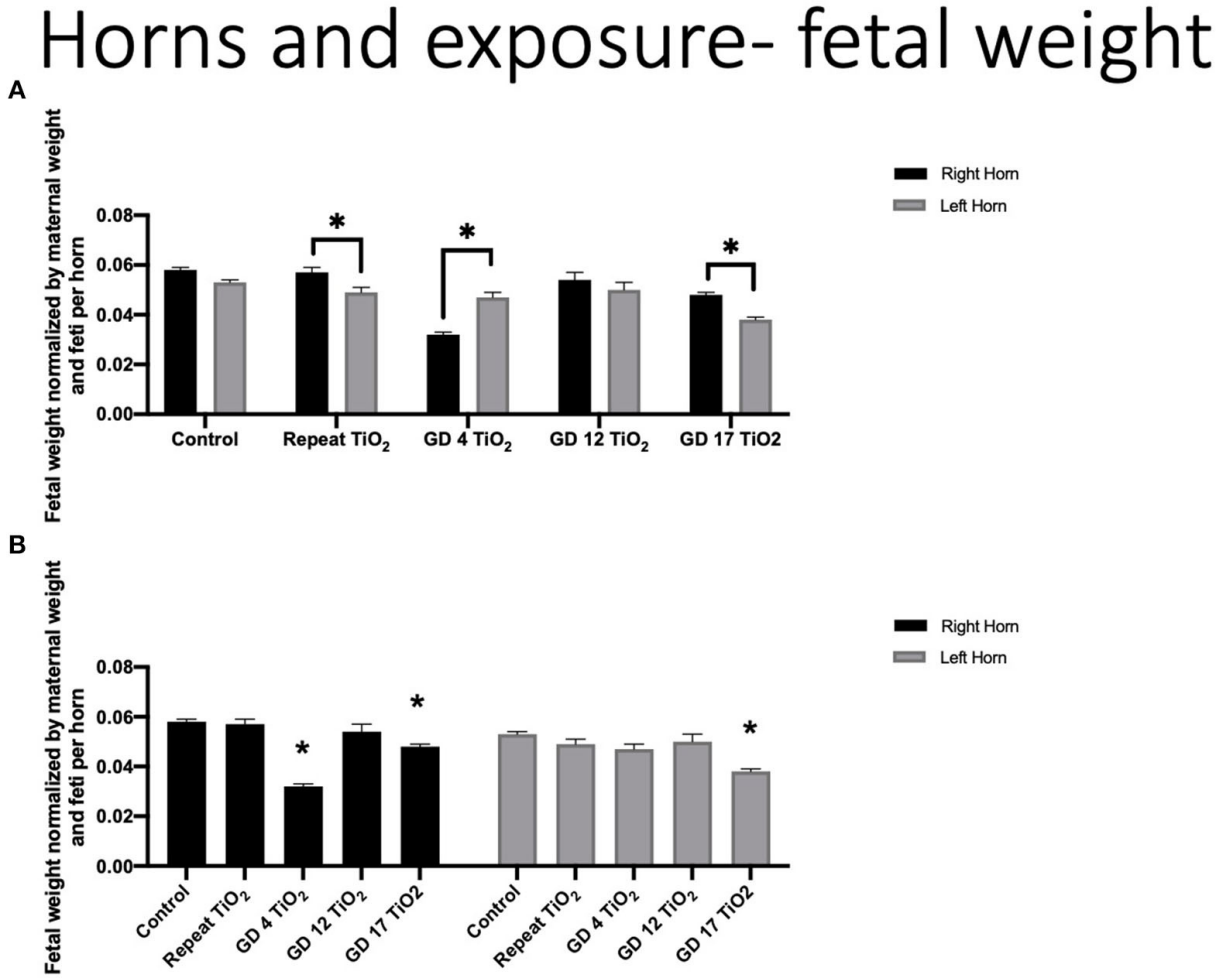
Fetal weight normalized by maternal weight on GD 20 and number of feti per uterine horn. **(A)** Normalized fetal weights comparing the right and left horn within each exposure timing condition. This analysis identifies differing fetal growth between the uterine horns within the same experimental condition. **(B)** Normalized fetal weight of the right and left horns for each exposure group compared to control. This analysis identifies fetal growth impaired by maternal exposure to nano-TiO_2_ during gestation. Significance is set to *p* < 0.05, indicated as * and values are shown as mean ± SEM.

Fetal weights were compared using our developed approach by organizing data by intrauterine position and normalizing by maternal weight at necropsy (GD 20) and number of feti per horn. In controls, average of total fetal weights from the left horn (0.053 g ± 0.001) were smaller than those in the right horn (0.058 g ± 0.001) (*p* = 0.08). When the overall right and left horn were compared for exposure groups, repeat and GD 17 exposure resulted in significantly smaller feti in the left horn (*p* = 0.02 and 0.01, respectively). GD 4 exposure resulted in significantly smaller feti in the right horn (*p* < 0.0001) (Figure 2A). GD 4 and GD 17 also produced significantly smaller pups (*p* < 0.0001 each) in the right horn, as compared to control growth in the right horn (Figure 2B). Comparisons for the left horn produced significantly smaller pups on GD 17 (*p* < 0.0001) (Figure 2B).

**Figure 3:**
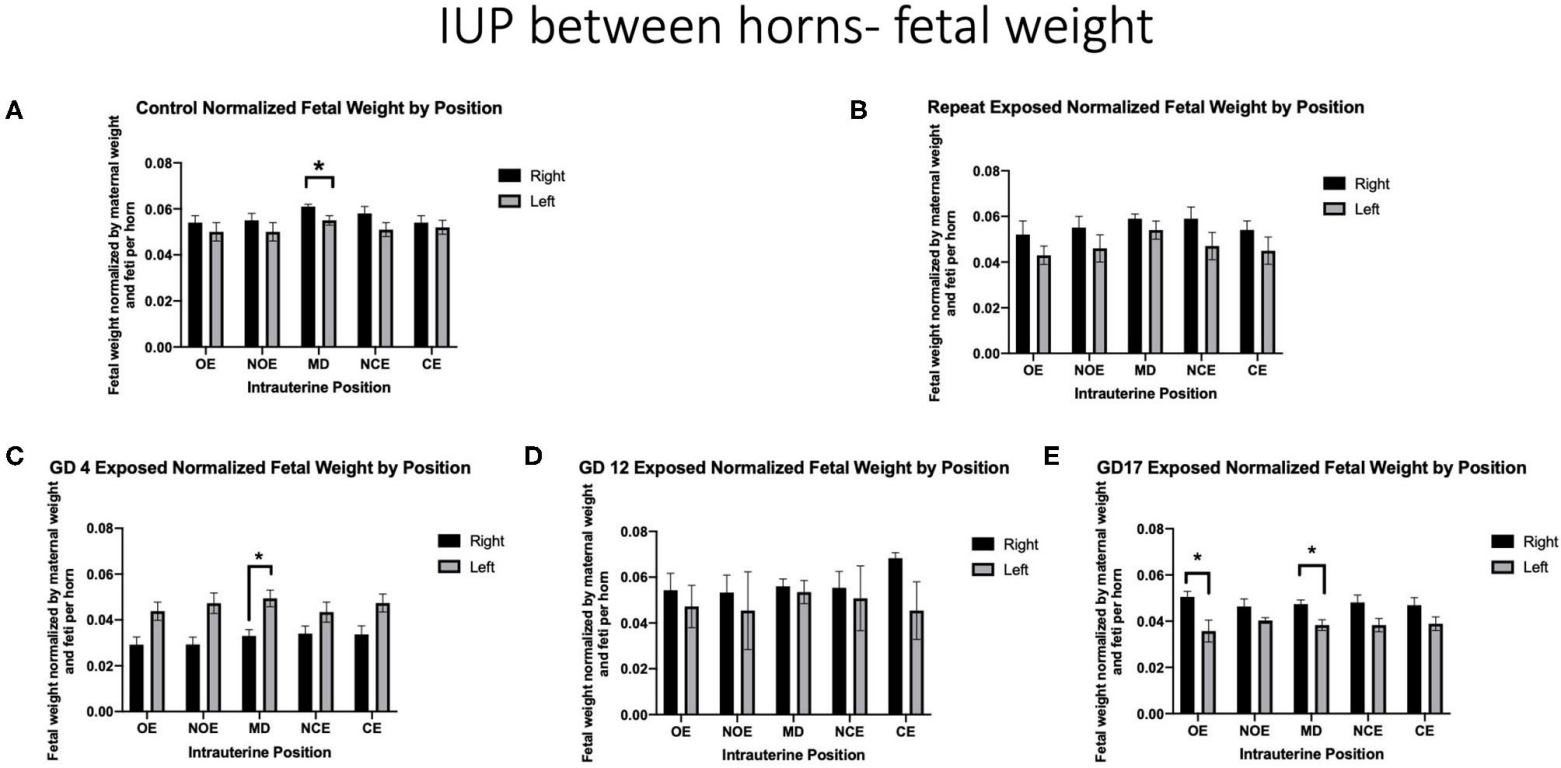
Fetal weight normalized by maternal weight on GD 20 and number of feti per horn. Data is analyzed by anatomical uterine position to identify differences between right and left uterine horns in **(A)** control and **(B–E)** exposure groups. Significance is set to *p* < 0.05, indicated as * and values are shown as mean ± SEM.

When positional outcomes (Figure 1) were included in the analyses, fetal weights were significantly smaller in the LMD position compared with the RMD position in controls (LMD 0.054 g ± 0.002 vs. RMD 0.060 g ± 0.001) (Figure 3A). The feti with the lowest weight in control dams were in the LOE and LNOE positions.

When analyzing fetal body weights, exposure to a single inhalation of nano-TiO_2_ during pregnancy had a differential effect on fetal weight by intrauterine location based on the timing of exposure. Dams that were repeatedly exposed had no position that was significantly impacted (Figure 3B). Dams exposed on GD 4 had significantly smaller fetal weight in the RMD location compared with the LMD location (0.033 g ± 0.003 vs. 0.049 g ± 0.004, respectively) (Figure 3C). Exposure on GD 12 resulted in no significant differences between intrauterine locations within and between right and left horns (Figure 3D). Damns exposed on GD 17 had significantly smaller feti on the LOE (0.036 g ± 0.005 vs. 0.051 g ± 0.002) and the LMD (0.038 g ±0.002 vs. 0.047 g ± 0.002) locations (Figure 3E).

**Figure 4:**
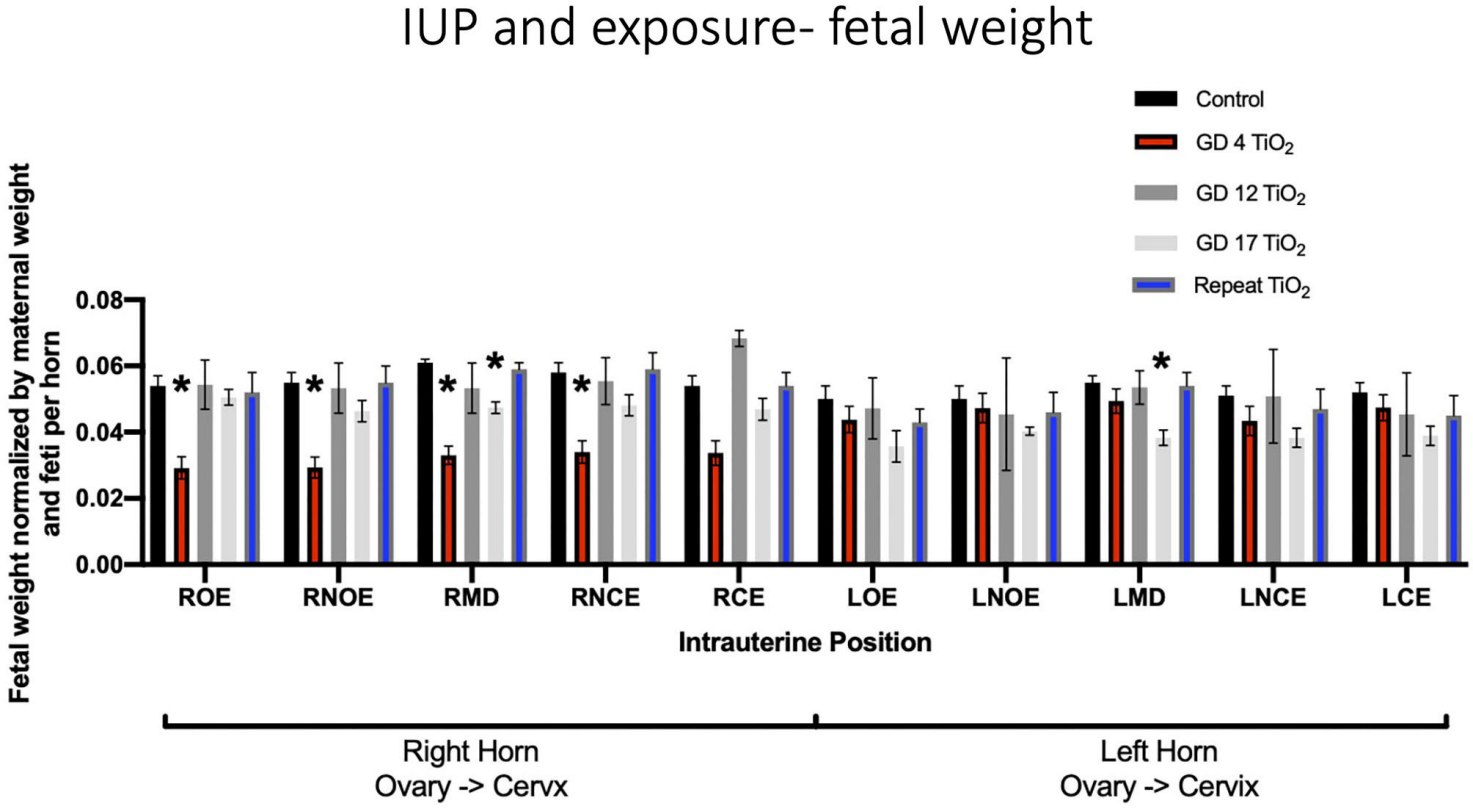
Fetal weight normalized by maternal weight on GD 20 and number of feti per horn. This analysis pinpoints intrauterine positions which are susceptible to growth reduction after certain timing of exposure(s) compared to controls. Significance is set to *p* < 0.05, indicated as *, and values are shown as mean ± SEM.

When comparing fetal weights between exposure groups on a per-location basis, exposure on GD 4 had a statistically significant impact on the ROE (0.052 g ± 0.003 vs. 0.029 g ± 0.003), RNOE (0.053 g ± 0.004 vs. 0.029 g ± 0.003), the RMD (0.060 g ± 0.001 vs. 0.033 g ± 0.003) and the RNCE (0.055 g ± 0.004 vs. 0.034 g ± 0.003) locations as compared to control (Figure 4). No significance was found between control and repeated exposed or control and GD 12 exposed groups at any intrauterine location. Exposure on GD 17 had a statistically significant impact, compared to control, on the LMD location (*p* = 0.003).

### Intrauterine Position Effect on Placental Weight

**Figure 5:**
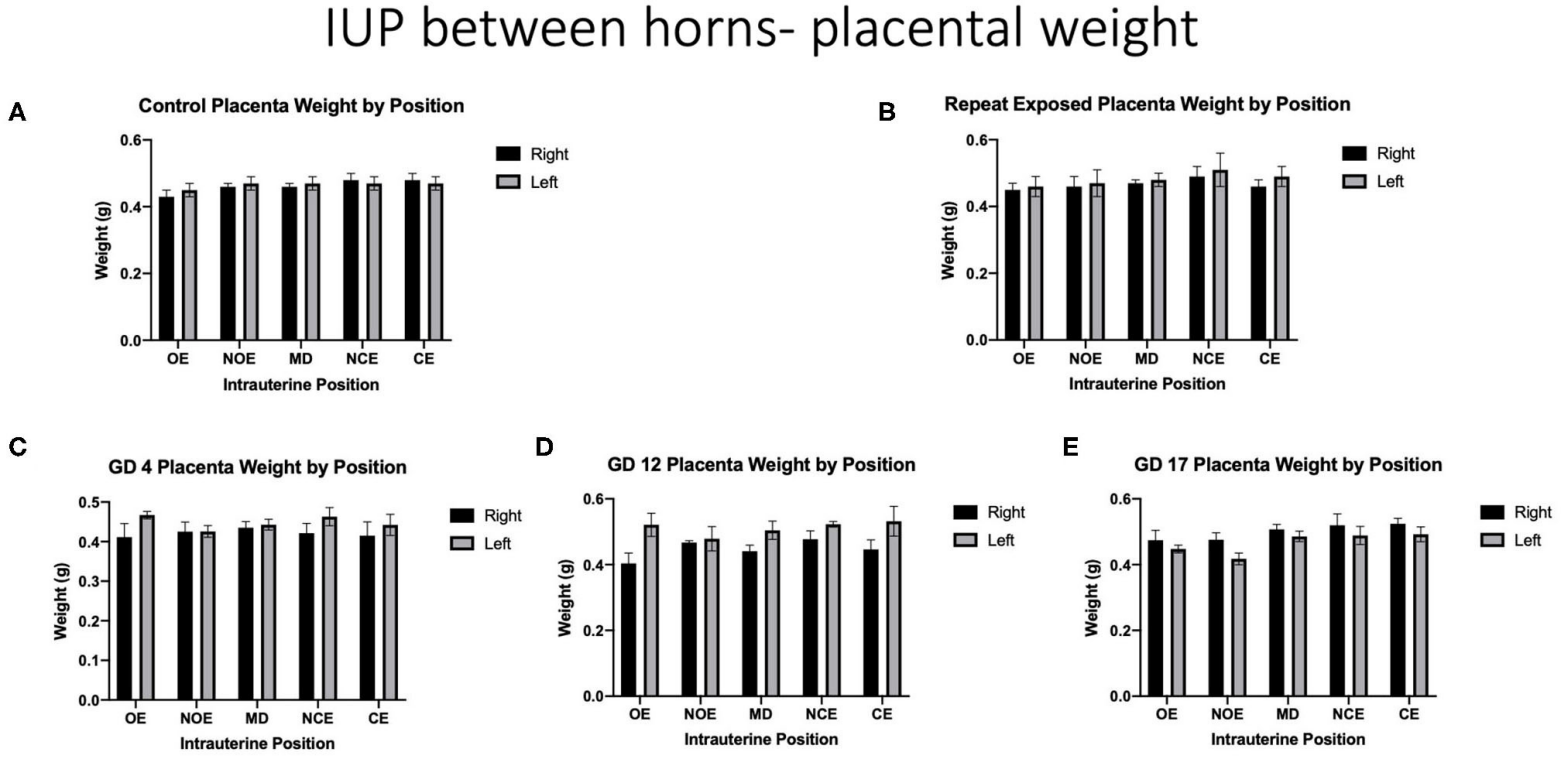
Raw placental weights (g). Data is analyzed by anatomical uterine position to identify differences between right and left uterine horns in **(A)** control and **(B–E)** exposure conditions.

**Figure 6:**
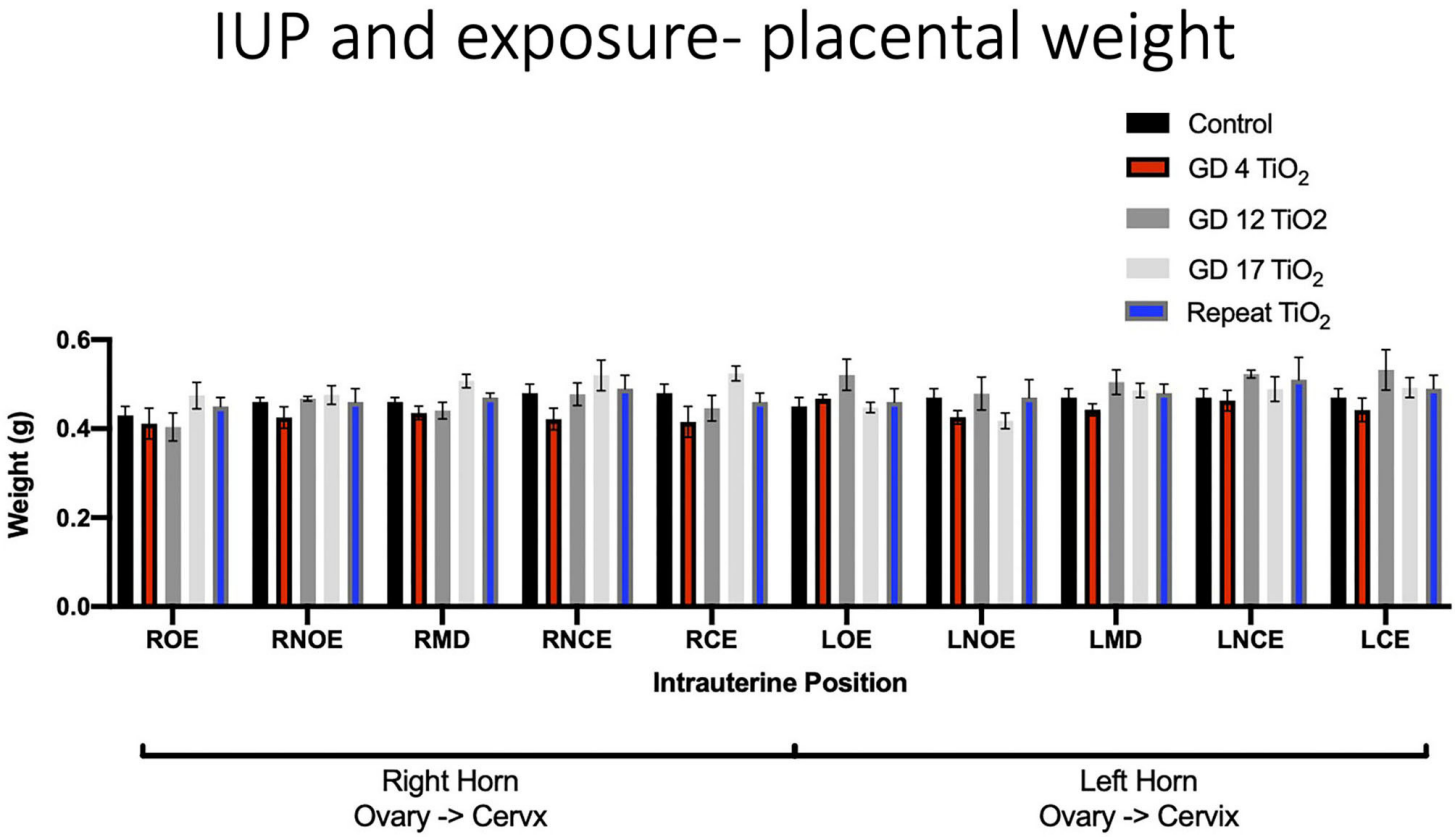
Raw placental weight (g) at each intrauterine position for control and exposure groups. This analysis pinpoints intrauterine positions which are susceptible to placental weight change after certain timing of exposure(s) compared to controls.

When analyzing for placental weight under control conditions, there was no significant impact on placental weight within and between the right and left uterine horns in control animals (Figure 5A). Placental weights were not impacted within or between intrauterine locations in the right and left uterine horns with repeated or single exposures to nano-TiO_2_ (Figures 5B–E). Comparison of raw placental weights at each intrauterine location between each experimental group and the control group showed no significance differences (Figure 6).

### Intrauterine Position Effect on Placental Efficiency

**Figure 7:**
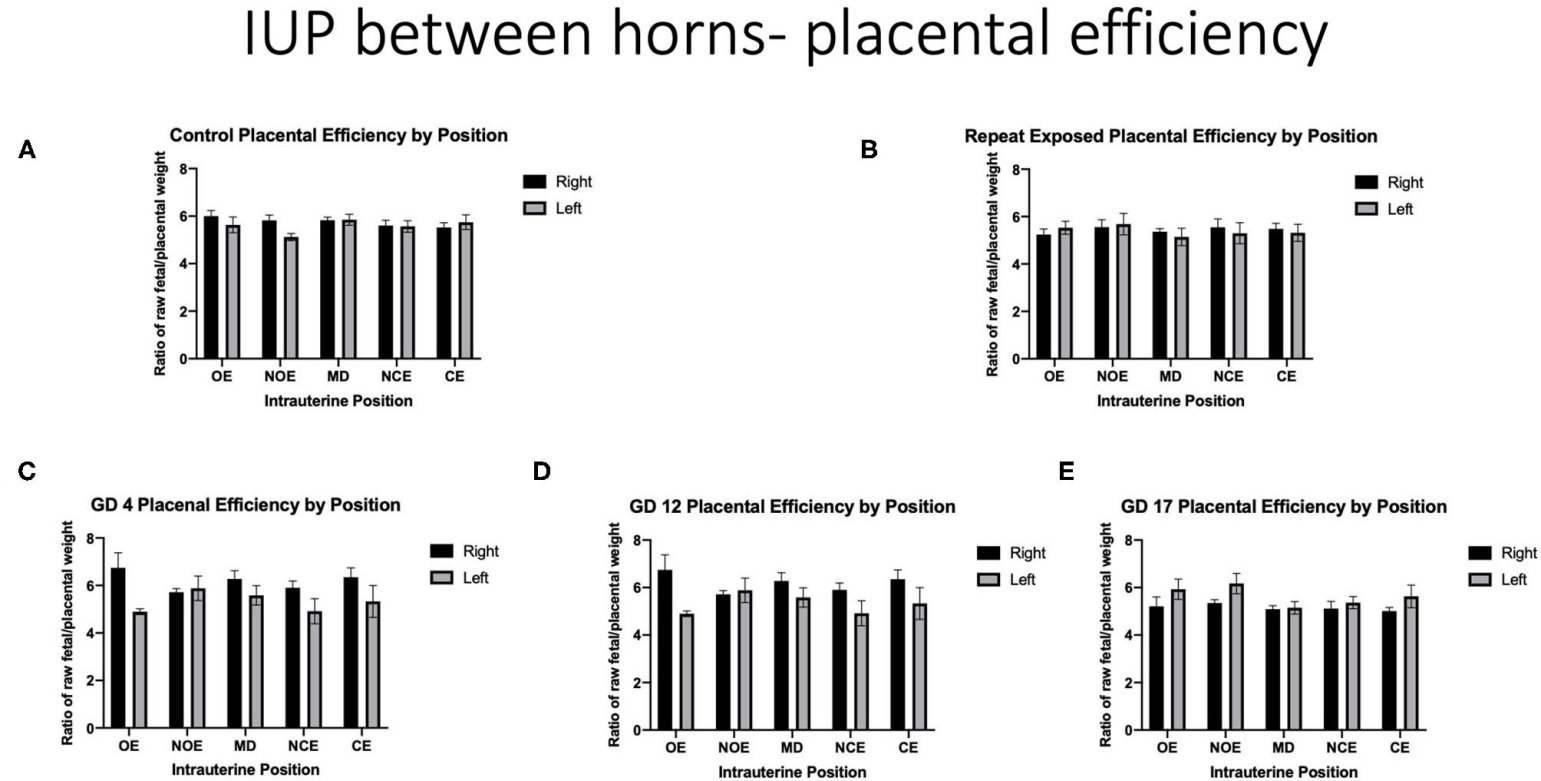
Placental efficiency calculated by fetal weight/placental weight. Data is analyzed by anatomical uterine position to identify differences between right and left uterine horns in control **(A)** and exposure groups **(B–E)**.

**Figure 8:**
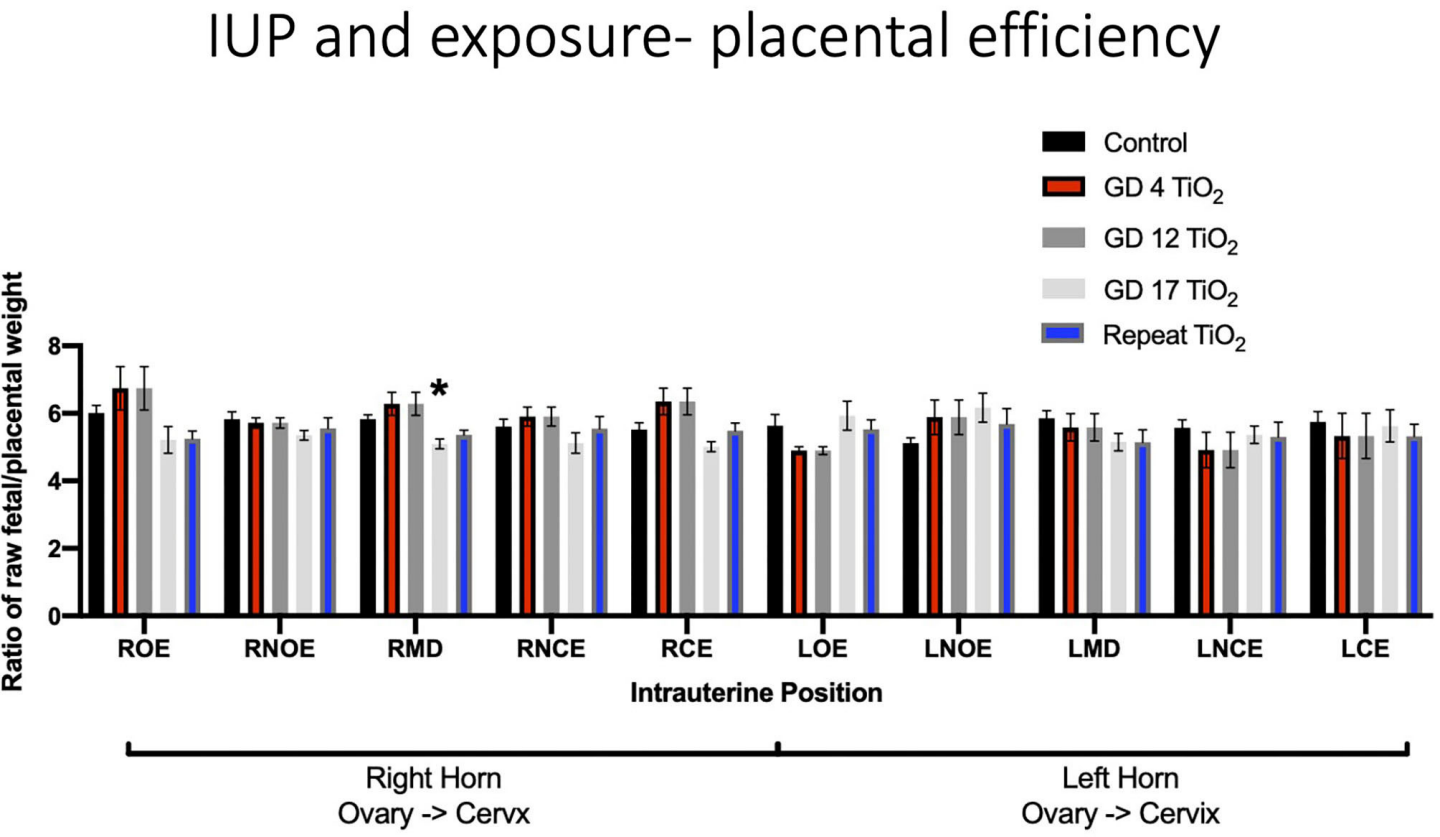
Placental efficiency, calculated by fetal weight/placental weight, for control and exposure groups. This analysis pinpoints intrauterine positions which are susceptible to reduced placental efficiency after certain timing of exposure(s) compared to controls. Significance is set to *p* < 0.05, indicated as *, and values are shown as mean ± SEM.

When analyzing for placental efficiency, the ratio of fetal weight to placental weight, under control conditions, there was no difference in either horn or at any location (Figure 7A). Placental efficiency was not impacted within and between intrauterine locations in the right and left uterine horns with repeated (Figure 7B) or single (Figures 7C–E) exposures to nano-TiO_2_ aerosols. When comparing placental efficiency between each experimental group and the control group no significance was found with the exception of a single exposure on GD 17 resulting in a significant reduction in placental efficiency at the RMD location (5.09 ± 0.14 vs. 5.74 ± 0.16) (Figure 8).

## Discussion

In this study, data from controls and experimental exposures to nano-TiO_2_ were evaluated in a manner that challenges the traditional dogma of litter data analysis. Herein, we were able to account for intrauterine position, number of feti per horn and maternal weight. When evaluating this dataset using traditional methods, we did not detect significant differences or more than 10% reduction in fetal growth between treatment groups. However, upon further evaluation using our revised approach we observed that under control conditions, feti implanted in the left uterine horn tended to be smaller in body weight at term compared with feti implanted in the right uterine horn. These analyses also demonstrated significant FGR in exposed animals compared with controls, separated by uterine horn and intrauterine position, outcomes that were lost with traditional approaches. This imbalance of fetal growth between the uterine horns was exacerbated after either repeated maternal exposure to nano-TiO_2_ aerosols during gestation or a single exposure early (GD 4) or in late (GD 17) pregnancy. The most severe FGR in terms of magnitude of impact and locations affected were apparent from a single maternal exposure on GD 4 in the middle position of the right uterine horn. The middle position of the right uterine horn was also impacted with respect to other endpoints including increased placental weight, reduced fetal weight, and decreased placental efficiency after a single exposure late in gestation, on GD 17. Interestingly, there was no significant positional impact after repeated maternal nano-TiO_2_ exposure compared to control in this cohort. Overall, this study demonstrates both critical windows of maternal exposure early and late in gestation and the risks associated with anatomical positioning of implanted fetus in the right horn. Moreover, this information was not gleaned using a traditional approach to data analysis, where no impact on fetal weight was detected.

Observations from control conditions are in agreement with findings from other studies, in that despite anatomical similarities, the right horn produced larger feti suggesting a more favorable environment than the left (Wiebold and Becker, 1987; Lan et al., 2010). This observation has also held from human studies, where there is a tendency for right ovary ovulation and implantation on the right side of the reproductive tract (Kawakami et al., 1993; Fukuda et al., 2000). The anatomical or physiological reasons behind this phenomenon are not understood. Moreover, when comparing each designated position between horns, a significant difference was found in the middle position. Others have reported the middle of the uterine horn to be impacted when considering intrauterine position in pigs (Perry and Rowell, 1969; Jang et al., 2014) and mice (Raz et al., 2012). Interestingly, rat studies have a species-specific effect where fetal growth is largest in the middle of each uterine horn (Jensh et al., 1970; Padmanabhan and Singh, 1981). This phenomenon may be explained by middle placentas receiving dual-artery blood supply from ovarian and uterine arteries (Avni et al., 2012). Our findings from the control group of animals are in agreement with these observations. In our experimental groups we observed feti in middle positions were also most susceptible to reduced body weight after exposure on GD 4 and GD 17 (Figures 3C,E). These findings suggest differences in fetal growth between intrauterine implantation positions under control conditions and after maternal exposure that may be applicable to DART studies.

When comparing between right and left horns (Figure 2) and specific intrauterine position within (Figure 3) after a single or repeated nano-TiO_2_ inhalation, the patterns of fetal growth were dependent on the timing of exposure during pregnancy. After a single exposure on GD 4, smaller feti were found in the right horn compared with the left, oppositional to all other findings (Figure 2A). Additionally, the middle position in GD 4 (Figure 3C) as well as GD 17 (Figure 3E) exposure fetal weights were impacted between horns (Figure 3). GD 17 exposed dams had an additional effect on the body weight of feti on the left horn (Figure 2B), and specifically in the ovarian end position (Figure 3E). Animals exposed repeatedly or on GD 12 did not present significant outcomes. These findings are also similar to other studies evaluating the gestational timing of maternal particulate inhalation exposures on FGR, wherein the outcome is dependent upon the pregnancy window(s) of exposure (Blatt et al., 2015; DeFranco et al., 2015).

The resulting FGR outcomes with maternal inhalation of nano-TiO_2_ may be associated with vascular dysfunction of the maternal uterine vascular tree. Previous studies have demonstrated a blunted relaxation and increased vascular smooth muscle contractility of the uterine artery following a single maternal exposure during early, mid, and late pregnancy (Fournier et al., 2019). Uterine microvascular dysfunction of the radial arteries has been identified in correlation with reduced fetal growth after repeated maternal nano-TiO_2_ inhalation in previous studies (Stapleton et al., 2013). Further, impaired basal arteriolar dilation was identified in late-stage pregnancy *in vivo* 24-hr after a single exposure to nano-TiO_2_ aerosols (Stapleton et al., 2018), unfortunately fetal growth was not evaluated in this study. The variability in fetal weight by location may be attributed to differences in perfusion of the uterine tissue, as previously demonstrated by arterial spin labeling MRI imaging in mice uterine vasculature (Raz et al., 2012). Dual-perfusion to the placental-fetal unit is delivered cranially from the ovarian artery and caudally from the uterine artery (Figure 1). In theory, there should be no change to blood quality or oxygenation between the ovarian, middle, or vaginal segments of the uterine horn (Burbank, 2012). However, as evidenced by human placental position studies and control animal data, balanced perfusion throughout the uterus may not be the case (Garris et al., 1983; Kalanithi et al., 2007; Zia, 2013; Jang et al., 2014; McLaurin and Mactutus, 2015). Raz et al. suggested feti at extreme ends of a uterine horn receive more nutrient- and volume-rich blood conveying a growth advantage over the middle positions in a normal pregnancy (Raz et al., 2012). They also suggest that this dual perfusion model may provide a survival advantage to fetus implanted in the middle of the uterine horn in cases of arterial perturbation (Raz et al., 2012). Other studies have shown non-uniformity in hematocrit dispersal, pressure gradients, and blood flow at vascular bifurcations, which may also occur at branching along the uterine arteries (Kalsho and Kassab, 2004; Sriram et al., 2014). During disease states, instances of non-uniformity or non-equitable blood distribution are exacerbated (Butcher et al., 2014; Frisbee et al., 2016); this may also be the case after environmental exposure as evidenced in Figure 2A. More research of blood distribution through the uterus and perturbations to uterine perfusion after maternal xenobiotic exposures is needed. Other potential mechanisms of toxicity may include: (1) impaired uterine angiogenesis (Bosquiazzo et al., 2010; Pereira et al., 2015), (2) increased uterine/spiral artery rarefaction (Baykal et al., 2004), (3) decreased quality of blood oxygenation or nutrition (Zamudio et al., 2010), (4) impaired placental function/nutrient transfer (Vrijer et al., 2004), (5) altered placental metabolism (Challis et al., 2000; Vaughan and Fowden, 2016), and (6) nano-TiO_2_ translocation culminating in physical blockade within the placenta (Ho et al., 2017; Zhang et al., 2018).

The placenta regulates the interface between maternal and fetal systems and is often implicated in cases of FGR as a reduction in placental efficiency. Interestingly, with the exception of GD 17 in the RMD position, neither placental weight nor efficiency were impacted. A change in placental weight may indicate placental stress and/or adaptation to the maternal milieu (Furukawa et al., 2011; Ouyang et al., 2013). Decreased placental efficiency ratios are associated with the development of chronic disease in later life, such as cardiovascular disease, diabetes, and other metabolic diseases (Martyn et al., 1996). Although we did not observe changes in placental weights and placental efficiency ratios that aligned with every pinpointed FGR location, placental oxidative stress balance and nutrient transfer may be perturbed at the molecular level (Huang et al., 2018; Zygula et al., 2020). Other environmental exposures, such as cadmium, are known to cause changes at the transcriptional level to the glucose transport proteins (GLUTs), impairing fetal glucose transfer (Xu et al., 2016). Alternatively, physical sedimentation of particles within the placenta may be causing physical blockage of fetal nutrient transfer (D'Errico et al., 2019; Fournier et al., 2020). Additional studies are required to understand potential placental molecular mechanisms that link exposure to nano-TiO_2_ to FGR outcomes.

The etiologies, pathogenesis, and potential therapies for FGR pregnancies have yet to be elucidated. Although many maternal factors (e.g., age, BMI, nutritional status, infections, cardiovascular, immune, pulmonary or thyroid disorders), administered medications [e.g., anticoagulants, steroids, beta blockers, anticonvulsants, and tranquilizers (Redmond, 1979)], and social behaviors [e.g., smoking, drinking, and substance abuse (Vogt Isaksen, 2004; Carter et al., 2013; Garrison et al., 2016; Sabra et al., 2017)] have been documented with human FGR studies, it is estimated that a large portion of FGR cases are of an unknown etiology. Domestic, occupational, or environmental exposures may play a large or confounding role in the initiation of FGR.

There are some limitations to our analyses in this study. While we focused on the effect of intrauterine growth positioning on fetal growth, the pathogenesis of FGR may also be influenced by fetal sex, a variable not assessed in these studies. Fetal sex plays a role in macronutrient uptake through the placenta, and thus sex-dependent differences in response to a growth-hindering intrauterine environment may occur (Mukhopadhyay et al., 2018). It has been hypothesized that male feti demonstrate a higher demand for nutrients for a rapid rate of growth; whereas, female feti are more frugal with nutrient uptake from the mother (Alur, 2019). Thus, because of this higher demand male feti may be more vulnerable to reduced growth if the maternal intrauterine condition is perturbed; in contrast, female feti may be more responsive to changes in the intrauterine environment, modifying growth to nutritional availability (Alur, 2019). Some studies that have stratified their fetal growth data by sex identified male-specific growth restriction after maternal conditions, such as gestational phthalate exposure (Zhao et al., 2014) and hypoxia (Thompson et al., 2020), have found evidence to support that FGR may be sex-dependent. Therefore, when conducting any assessment for fetal growth, stratifying data by fetal sex will be important to reveal sex dependent FGR. Future studies should include this variable in their analyses. Second, maternal body weight pre-pregnancy (Zhao et al., 2018) and gestational weight gain (Ludwig and Currie, 2010; Sato and Miyasaka, 2019) have been shown to correlate with fetal growth. Studies show larger females deliver larger feti, reasonably a result of more nutrition available for fetal growth. In this study maternal body weight on GD 20 was recorded and used in normalization calculations. Future studies should utilize maternal weight gain throughout pregnancy (e.g., maternal weight prior to implantation and at sacrifice or delivery) to have a better representation of exposure effects and nutritional concentrations available for fetal growth.

The overall goal of DART is to use information gained from animal studies to identify hazardous substances for a developing fetus. It is of utmost importance that reliable results are generated from these investigations; disregard for disparities associated with intrauterine positioning may lead to future studies riddled with variability. Therefore, clarity on the intrauterine positioning of feti such as those utilized and analyzed in these studies is paramount. Herein, we presented evidence of anatomical intrauterine positional effects after maternal exposure to nano-TiO_2_ aerosols, thereby challenging common DART dogma. Reconsidering the way in which these data are evaluated may reduce study variability and allow for more refined conclusions of hazard to fetal growth to be drawn.

## Data Availability Statement

The raw data supporting the conclusions of this article will be made available by the authors, without undue reservation.

## Ethics Statement

The animal study was reviewed and approved by Rutgers University Institutional Animal Care and Use Committee.

## Author Contributions

JD'E was responsible for the chronic animal exposures, data organization and analysis, and writing of this manuscript. SF was responsible for single animal exposures and critical review of the manuscript. This manuscript was conceptually attributed to and written under the guidance of PS. All authors contributed to the article and approved the submitted version.

## Conflict of Interest

The authors declare that the research was conducted in the absence of any commercial or financial relationships that could be construed as a potential conflict of interest.

## Supplementary Material

Supplementary Table 1 Table of effect sizes for traditional analysis approach (T-Test) and for our developed method (2-Way ANOVA).

## Abbreviations

DART: Developmental and Reproductive Toxicology
FGR: Fetal Growth Restriction
GD: Gestational Day
GLUT: Glucose Transporter Protein
IUP: Intrauterine Position
Nano TiO_2_: Titanium Dioxide nanoparticles
R/L CE: Right/Left Cervical End
R/L MD: Right/Left Middle
R/L NCE: Right/Left Next-to Cervical End
R/L NOE: Right/Left Next-to Ovarian End
R/L OE: Right/Left Ovarian End
SD: Sprague Dawley
SMPS: Scanning Mobility Particle Sizer.
