# Supplementary material for: Considering Intrauterine Location in a Model of Fetal Growth Restriction After Maternal Titanium Dioxide Nanoparticle Inhalation
Source: Front Toxicol. 2021 Mar 23;3:643804. doi: 10.3389/ftox.2021.643804 (PMC8121264; doi:10.3389/ftox.2021.643804)
Supplement: Supplementary Table 1 — Table of effect sizes for traditional analysis approach (T-Test) and for our developed method (2-Way ANOVA). [file Table_1.pdf]

Supplemental Table 1.

Traditional, T-Test	P-value	Effect Size (Cohen's d)
Repeat	0.13	0.57
GD 4	0.15	0.66
GD 12	0.87	0.07
GD 17	0.55	0.24

Effect Level Threshold:
Small= 0.2
Medium= 0.5
Large= 0.8

IUP, 2-Way AOVA	DF	F	P-value	Effect Size
**Control (Left and Right Horn)**				
IUP	4	F (4, 207) =0.2387	0.02	0.05
**Repeat**				
IUP	4	F (4, 97) = 1.675	0.16	0.01
**GD 4**				
IUP	4	F (4, 51) = 0.3500	0.84	0.02
**GD 12**				
IUP	4	F (4, 43) = 0.2270)	0.92	0.02
**GD 17**				
IUP	4	F (4,64) = 0.01026	0.99	0.0005
**Exposure and IUP**				
IUP	9	F (9, 451) = 1.488	0.15	0.02
**Exposure and Horn**				
Timing of Exposure	4	F (1, 503) = 4.086	0.004	0.1818

Effect Level Threshold:
Small= 0.01
Medium= 0.059
Large= 0.138
